# Bovine Colostrum and Its Potential for Human Health and Nutrition

**DOI:** 10.3389/fnut.2021.651721

**Published:** 2021-06-21

**Authors:** Ayşenur Arslan, Merve Kaplan, Hatice Duman, Ayşe Bayraktar, Melih Ertürk, Bethany M. Henrick, Steven A. Frese, Sercan Karav

**Affiliations:** ^1^Department of Molecular Biology and Genetics, Canakkale Onsekiz Mart University, Canakkale, Turkey; ^2^Uluova Dairy, Canakkale, Turkey; ^3^Evolve Biosystems, Inc. Davis, CA, United States; ^4^Department of Food Science and Technology, University of Nebraska Lincoln, Lincoln, NE, United States; ^5^Department of Nutrition, University of Nevada Reno, Reno, NV, United States

**Keywords:** bovine colostrum, human health, bioactive proteins, oligosaccharides, infants

## Abstract

Colostrum is the first milk produced post-partum by mammals and is compositionally distinct from mature milk. Bovine colostrum has a long history of consumption by humans, and there have been a number of studies investigating its potential for applications in human nutrition and health. Extensive characterization of the constituent fractions has identified a wealth of potentially bioactive molecules, their potential for shaping neonatal development, and the potential for their application beyond the neonatal period. Proteins, fats, glycans, minerals, and vitamins are abundant in colostrum, and advances in dairy processing technologies have enabled the advancement of bovine colostrum from relative limitations of a fresh and unprocessed food to a variety of potential applications. In these forms, clinical studies have examined bovine colostrum as having the substantial potential to improve human health. This review discusses the macro-and micronutrient composition of colostrum as well as describing well-characterized bioactives found in bovine colostrum and their potential for human health. Current gaps in knowledge are also identified and future directions are considered in order to elevate the potential for bovine colostrum as a component of a healthy diet for a variety of relevant human populations.

## Introduction

Colostrum is the earliest milk produced from the mammary glands for the first few days after giving birth and is unique in its composition of essential nutrients, immune factors, and oligosaccharides that benefit the newborn (1, 2). In the case of cows, bovine colostrum is produced immediately after calving and quickly wanes to mature milk (3), which lacks the high level of beneficial nutrients found in bovine colostrum. There are several factors affecting the composition and physical properties of colostrum such as individuality, breed, parity, pre-partum nutrition, length of the dry period of cows, and time post-partum (4). Generally, colostrum has more fat, protein, peptides, non-protein nitrogen, ash, vitamins and minerals, hormones, growth factors, cytokines, nucleotides, and less lactose compared to mature milk content. The concentration of these compounds decreases rapidly in the first 3 days of lactation with the exception of lactose content (5–7).

While the consumption of human colostrum by infants has long been recognized as a source of critical bioactive proteins for infants (8), the consumption of animal colostrum is also practiced in many locations beyond the neonatal period (9, 10). In these cultures and regions, colostrum has long been consumed as a health food or for medicinal purposes, with cultural practices centered on the belief that animal colostrum was an important component of the development of healthy children and supportive of healthy or infirmed adults (9, 11, 12). While these cultural or regional beliefs are associated with this practice, the abundance of well-characterized bioactive compounds and selective prebiotic components of this food may further support this cultural knowledge from a scientific perspective.

Historically, liquid fresh colostrum was primarily consumed, but pasteurized colostrum is also commercially available as a standalone drink, though production remains small (13). In European cultures and elsewhere such as India, and Scandinavia, colostrum is also used in the production of cheeses and other traditional foods (14). More recently, dried colostrum is collected and processed as a dietary supplement, which is widely consumed for perceived health benefits (10). In the US and EU, colostrum supplements are marketed for a variety of health benefits, including boosting immunity and gastrointestinal (GI) health. While attractive in concept, there are limitations to this use of dried colostrum, which are typically in a pill or tablet form, given the limited amount of colostrum consumed relative to clinically studied consumption rates.

Still, colostrum is a complex biological fluid and contains significant components which are natural anti-microbial factors for stimulating the maturation of calf immunity (15). In addition, the development and function of the GI tract are shaped by colostrum intake (5, 6, 16–18), and it also affects the metabolic and endocrine systems as well as the nutritional state of neonatal calves (5, 6, 17). Colostrum has muscular-skeletal repair and growth potential in addition to its immune support function and many benefits to health because of its content of bioactive proteins (19). Further, some evidence suggests that the cytokines, immunoglobulins, growth factors, antimicrobial compounds, and maternal immune cells are transferred to the newborn with the feeding of colostrum to support neonatal immunity (20–22). Bovine colostrum has even been purported to treat viral and bacterial infections as a nutraceutical (23). Together, the existing evidence in support of colostrum suggests that there is potential for colostrum to have a significant role in supporting human health as well. While there are other studies which have begun to look at colostrum from other animals (24–27), this review explores the current knowledge on the bovine colostrum in the context of nutrition, its bioactive components, and its potential for human health and nutrition.

### Bovine Colostrum Composition

Milk composition changes dramatically over the course of lactation and bovine colostrum is compositionally and nutritionally distinct from mature milk (28). In contrast to mature milk, colostrum has a much higher protein and moderately higher fat content, with substantially less lactose (Table 1). This reflects the needs of the developing calf, where the passive transfer of immunoglobulins is critical for health (41).

**Table 1:** Bovine colostrum and mature milk composition.

**Colostrum component**	***n*^a^**	**Mean**	**Minimum**	**Maximum**	**SE**	**Mature milk**
**Bovine colostrum**						
Fat mg/mL	1,226 (29)	64.00	41.00	83.00	33.20	39.00 (28)
	54 (30)	67.00	20.00	265.00	41.60	
Protein mg/mL	1,226 (29)	140.00	116.00	166.00	36.70	36.00 (28)
	55 (30)	149.20	71.00	226.00	33.20	
Casein mg/mL	– (31)	43.00	–	–	–	25.00 (31)
Whey mg/mL	– (31)	120.00	–	–	–	5.10 (31)
Lactose mg/mL	1,226 (29)	27.00	23.00	31.00	5.50	49.00 (28)
	55 (30)	24.90	12.00	52.00	6.50	
Dry matter mg/mL	55 (30)	276.40	183.00	433.00	58.40	125.00 (28)
Ash mg/mL	55 (30)	0.50	0.20	0.70	0.10	7.00 (28)
IgG mg/mL	1,239 (29)	55.00	38.10	67.80	25.75	0.257 (32)
IgA mg/mL	55 (30)	1.66	0.50	4.40	0.50	0.04–0.06 (12, 30, 33, 34)
IgM mg/mL	55 (30)	4.32	1.10	21.00	1.10	0.03–0.06 (12, 30, 33, 35)
Oligosaccharides mg/mL	– (36)	–	0.70	1.20	–	0.3–0.5 (36)
Lactoferrin mg/mL	55 (37)	0.82	0.10	2.20	0.10	0.10–0.30 (37)
Lactoperoxidase mg/mL	– (38)	–	11.00	45.00	–	13–30 (38)
Ca mg/kg	55 (30)	4,716.10	1,898.00	1,775.10	8,593.50	1,220.00 (39)
	– (40)	1,518.60	–	–	–	
P mg/kg	55 (30)	4,452.10	1,706.29	1,792.40	8,593.5	1,520.00 (39)
	– (40)	1586.00	–	–	–	
Mg mg/kg	55 (30)	733.24	286.07	230.30	1,399.60	120.00 (39)
	– (40)	219.70	–	–	–	
Na mg/kg	55 (30)	1,058.93	526.02	329.70	2,967.80	580.00 (39)
	– (40)	516.70	–	–	–	
K mg/kg	55 (30)	2,845.89	1,159.89	983.20	5,511.40	1,520.00 (39)
	– (40)	1,297.50	–	–	–	
Zn mg/kg	55 (30)	38.10	15.90	11.20	83.60	5.30 (39)
	– (40)	151.00	–	–	–	
Fe mg/kg	55 (30)	5.33	3.09	1.70	17.50	0.80 (39)
		34.66	–	–	–	
Mn^b^ mg/kg	23 (30)	0.10	0.11	0.00	0.36	0.20 (39)
	– (40)	2.62	–	–	–	
Vitamin A mg/kg	55 (30)	4.90	1.82	1.40	19.30	460.00 (39)
Vitamin E mg/kg of fat	55 (30)	77.17	33.52	24.20	177.90	2.10 (39)
Vitamin B12 μg/mL	5 (30)	0.60	0.35	0.20	1.10	4.50 (39)

a *Number of colostrum samples reported in the referenced study*.

b *Part of the samples were quantified as <0.05 and therefore not included in averages*.

Further, as the volume of milk production increases over lactation, there is a concomitant decrease in the mineral content of milk (Table 1). Thus, colostrum represents a relatively high-protein and lower-carbohydrate solution that can be processed similarly to mature milk in order to reduce fat content and shape the caloric density for desired nutritional applications. Further, milk proteins are considered a “complete protein” source owing to their amino acid profile, and high protein digestibility, especially of whey proteins (42), though colostrum contains higher concentrations of immunoglobulins which are less digestible (Table 1).

While current dietary recommendations of protein intake for a healthy adult with minimal physical activity are 0.8 g per kg per day (43, 44), a growing body of evidence suggests that optimal intake may be higher [1.2–1.6 g per kg per day; (45–47)] and this intake should be balanced across meals to promote skeletal muscle protein synthesis (48). Especially in elderly populations, optimal protein intake to reduce skeletal muscle loss associated with aging is often not achieved, which is further compounded by diminished proteolytic activity associated with aging (49). Thus, colostrum may offer an attractive digestible, complete protein source that can be integrated into a calorically-appropriate diet. In addition to macronutrients, bovine colostrum includes vitamins, minerals, and a broad assortment of protein-derived bioactives which may offer additive benefits to its macronutrient profile.

#### Main Factors Affecting Colostrum Composition

The composition and quality of the bovine colostrum are highly variable due to genetic and environmental factors including individuality, breed, parity, the timing of milking, diseases, pre-partum nutrition, season, length of the dry period of cows, and time post-partum (50–52).

##### Individual Variation Among Animals

Bovine colostrum quality is different among individuals and between genetic backgrounds (31). For instance, the concentration of immunoglobulin G (IgG) in bovine colostrum and the volume of first milking vary among individual cattle (53, 54). The Jersey cows produce the highest (66.5 g/L) whereas Friesian-Holsteins produce the lowest (41.2 g/L) concentrations of IgG among breeds studied (55). In the case of cow parity, first-calf heifers produce a lower yield of colostrum and lower IgG concentration in colostrum than those cows in their second or greater lactation. The quality of bovine colostrum increases with parity after the second calving, and older cows generally produce the best quality colostrum (54).

Another individual factor is the disease which influences bovine colostrum quality. For instance, mastitis is an inflammation of the mammary gland of the bovine that has negative consequences including low quality of the colostrum. The volume and concentration of bovine colostrum IgG are lower in cows with infected mammary glands than cows with uninfected glands (56). The age of cows also affects the quality of colostrum. Some studies' data are in general agreement that older cows have a higher quality of colostrum than younger cows (53, 54, 57). The association between older age and good quality of colostrum is thought to be a result of increased pathogen exposure, improved immunity, and body condition score (31).

##### Environmental Factors

The timing of the bovine colostrum milking after parturition has significant effects on concentrations of IgG in the bovine colostrum. Early or immediate colostrum milking will significantly increase colostrum quality. Moore et al. (58) reported that colostrum collected 6, 10, and 14 h after parturition has lower IgG concentration than colostrum collected 2 h after parturition. Another study also showed that bovine colostrum quality is highest immediately after parturition of North American herds, but it decreased when milking was delayed (53, 58). Bovine colostrum quality is also affected by the calving season. Cows calving during the summer months have lower quality colostrum than those calving in the autumn months (53). The bovine colostrum fat percentage is at 24 and 48 h after birth is affected by the calving season. Animals born in autumn-winter seasons have a higher colostrum fat percentage than those in calving in spring-summer seasons. One cause may be differences in metabolism, feed, and water consumption in different seasons (59, 60).

The dry period length is an important period for cows which lasts ~6–8 weeks. This period is needed for the renewal of milk secretion tissue, preparation for lactation, and completion of fetus development (61–63). Colostrum starts to be secreted in the last 15–20 days of the dry period and its composition changes continued until parturition (62, 64). Le Cozler et al. (65) also reported that there is a positive coefficient of correlation (R^2^ = 0.22; *P* < 0.01) between IgG concentration and dry period length (65).

### Fats

Colostrum contains a higher percentage of fat than mature milk (66) and the composition of these fats is also distinct. O'Callaghan et al. (67) examined the composition of colostrum and the changes observed during the transition to mature milk, reporting that colostrum is higher in palmitic, palmitoleic, and myristic acids, relative to mature milk (67). While these fat profiles are well-suited to the developing calf (68), the profiles of these fats and the higher concentration of saturated fat have been associated with long-term negative health outcomes, though there is some disagreement within the literature as to the level of support for the role of dairy fats in cardiovascular disease (69). There is evidence that these fatty acids play a role as signaling molecules and, as dietary fatty acids, contribute to the regulation of lipogenesis in the liver (70). Further, many vitamins found in milk are fat soluble (e.g., vitamin A, D) and removal of these fats also reduces the concentration of these vitamins in colostrum.

It is of relevance to consumers that the advances in dairy technology which enable efficient separation of fats from the aqueous fraction of milk (that is, the fraction which contains proteins, carbohydrates, minerals, and some vitamins) enable the reduction or removal of these fats from colostrum, ahead of downstream processing, making the potential for a low-fat or fat-free colostrum product possible. However, some have speculated that the tradeoff between dairy fats and the removal of bioactive found in the fat fraction of dairy foods may not always be a net benefit (69). To resolve this conflict in the literature, it is clear that well-controlled clinical studies investigating the relationship between the dietary fats found in colostrum and health are needed.

### Vitamins/Minerals Found in Colostrum

Bovine colostrum contains also high levels of fat-soluble and water-soluble vitamins that are critical to human health (4). Notably, vitamin A is reported to be found at high concentrations in colostrum in a variety of forms including retinol, retinal, retinoic acid, retinyl esters and as provitamin A carotenoids (71–73). Vitamin E, in the form of tocopherols and tocotrienols (~ mean 77.17 mg/kg) are found in low density lipoproteins in colostrum (4, 30). Vitamin K is also found in greater concentration in colostrum compared to mature milk in two forms, phylloquinone, and menaquinones (71). Vitamin D is found in higher concentrations in colostrum than mature milk (74). Vitamin D has important roles in immune activities and promotes the uptake of calcium and phosphorus in the small intestine (75). It has two major forms as cholecalciferol (vitamin D3) and ergocalciferol (vitamin D2) and their concentration decreases from 1.2 to 0.36 IU. g−1 during the first 5 days post-partum (76). Vitamin C and the B vitamins are also found in the water-soluble fraction of colostrum at a higher concentration compared to mature milk (77) and together provide a natural source of essential vitamins critical to human health.

Bovine colostrum and mature milk are known to be good sources of several minerals especially calcium and phosphorus (75). Recent studies revealed that the mean concentrations of several important minerals in colostrum are significantly higher than in mature bovine milk. Calcium is necessary for the maintenance of calf development and their healthy bones and teeth. Phosphorus is also crucial for the metabolic rate and physiological functions including development of skeletal tissue, energy utilization, protein synthesis, and transport of fatty acids (78). Magnesium is present in a relatively large amount, along with zinc and selenium in bovine colostrum (75).

### Bioactive Proteins

#### Immunoglobulins (Igs)

Immunoglobulins (Igs) are complex proteins, known as antibodies, that make up a significant part of the total protein in bovine colostrum. The immunoglobulins in bovine colostrum mainly come in 3 different varieties called isotypes including IgG (IgG1 and IgG2), IgA, IgM. IgG is the dominant immunoglobulin in bovine colostrum, which makes up 85–90% of the total immunoglobulin content. IgG1 represents 80–90% of the total IgG content in bovine colostrum, followed by IgM, IgA, and IgG2 (23, 79, 80). These immunoglobulins are essential in the survival of the calves and their immune systems and they neutralize enteric pathogens such as bacteria, microbes, and viruses. Using bovine colostrum as a source of antibody preparations to support bovine and human health is an important research subject that has been studied for decades (81).

One of the key differences between mature milk and colostrum is the high concentration of IgG found in colostrum, which reaches up to 50–100 mg/mL in the first days after birth (33, 82, 83). Bovine serum IgG1 and IgG2 concentration decrease before parturition, they are transferred from the blood into the colostrum. In fact, nearly all IgG in colostrum is transferred from bovine serum into the colostrum and milk (84, 85).

The high concentration of IgG is necessary for the survival of calves, which is strongly dependent on the transfer of IgG from bovine colostrum to calves to provide passive immunity as cows cannot transfer IgG through the placenta (86). Indeed, if calves do not receive colostrum immediately after birth, they are prone to infection and will suffer from a higher risk of morbidity and mortality (31, 87, 88).

#### Lactoferrin

Lactoferrin is a cationic, iron-binding glycoprotein present as about 0.80 mg/mL in bovine colostrum (37). It has multiple functions including antibacterial, antifungal, antiviral, antiparasitic, antitumor and immunomodulatory (anti-inflammatory) effects (23, 35, 89, 90), and is the major protein in the milk serum of all mammals (91). Bovine colostrum-derived lactoferrin has antimicrobial activity by inhibiting the growth of disease-causing protozoa, yeasts, bacteria, and viruses, and lactoferrin may prevent the attachment of pathogens to epithelial cells and help maintain intestinal permeability and stability (83, 92, 93). Moreover, there are some studies showing that bovine colostrum-derived lactoferrin can increase the proliferation of cells involved in the bone formation such as osteoblasts, and the release of some growth factors from osteoblasts (94, 95).

Furthermore, it is known to play a role in iron uptake in the intestine and activation of phagocytes and immune responses. Receptors for lactoferrin are expressed on intestinal tissue, monocytes, macrophages, neutrophils, lymphocytes, platelets, and on some bacteria (96). Bovine lactoferrin supplements are thought to support the immune system and influence immune cell activity potentially via these antioxidant, antibacterial, and antiviral properties (97). The greatest concentration of this protein is found in colostrum, which has been determined to be four times greater than mature milk (98).

#### Lactoperoxidase

Lactoperoxidase is a major antibacterial enzyme found in bovine colostrum, it is a basic glycoprotein that catalyzes the oxidation of thiocyanate and generates intermediate compounds with antimicrobial activities (99). The concentration of lactoperoxidase is 11–45 mg/L in bovine colostrum and 13–30 mg/L in mature bovine milk (38). Its concentration in bovine colostrum is low initially, but it reaches the maximum level within 3–5 days after parturition. Lactoperoxidase catalase activity is also higher in bovine colostrum than in mature milk (100, 101).

Lactoperoxidase activity produces toxic oxidation products that inhibit bacterial metabolism by oxidation of essential sulfhydryl groups in proteins. This system is toxic to some gram-positive and negative bacteria like *Pseudomonas aeruginosa, Salmonella typhimurium, Listeria monocytogenes, Streptococcus mutans*, and *Staphylococcus aureus* (102). The lactoperoxidase system also inactivates the poliovirus, vaccinia virus, and HIV (93, 103, 104).

### Oligosaccharides

Bovine colostrum is a rich source of complex and highly selective oligosaccharides and glycans. The concentration of oligosaccharides in colostrum is 0.7–1.2 mg/mL and the majority of these structures are acidic oligosaccharides which are lower in mature bovine milk (36, 105). Forty distinct oligosaccharides compositions have been detected in bovine colostrum so far (106–108). The total colostrum oligosaccharides differ between cows because of their genetic variability (109). Predominant oligosaccharides in bovine colostrum are 3′ sialyllactose (3′SL), 6′ sialyllactose (6′SL), 6′ siayllactosamine (6′SLN) and disialyllactose (DSL). 3'SL is 70% of total oligosaccharide content in bovine colostrum (105, 107, 110, 111). 3′SL, 6′SL, and 6′SLN levels in colostrum were highest following parturition and decreased by 48 h post-partum, while neutral oligosaccharide level increased (105). Breed specific differences have also been identified in oligosaccharide content. Concentrations of 3′SL, 6′SL, 6′SLN and DSL were found as 867, 136, 220, and 283 μg/mL, respectively, in colostrum from Jersey cows, while these concentrations were 681, 243, 239, and 201 μg/mL, respectively, in Holstein colostrum after parturition (112). Both free oligosaccharides (bovine milk oligosaccharides, BMOs) and complex, conjugated *N*-glycans represent the majority of the prebiotic components of bovine colostrum (113).

While there are many distinctions between BMOs and human milk oligosaccharides (HMOs), there has been significant interest in utilizing milk and colostrum as a source of BMOs for human nutrition and health to modulate the GI microbiome (114). In contrast to HMOs, BMOs are predominantly sialylated (i.e., acidic) oligosaccharides, with a low propensity for fucosylation (106) and a lower structural diversity (106). Recent advancements in enzymatic glycosylation have provided opportunities for the structural enhancement of BMOs to alter their structure to resemble HMOs (115). Several complexities in milk processing have thus far limited the ability of BMOs to be separated from lactose found at high concentrations in milk (114), though solutions have begun to emerge (116) which complicates their utility for human nutrition and health. Further, though pilot experiments with purified BMOs in adults have not yet demonstrated generalizable changes to GI microbial populations (117), future work in infants may be more promising as recent *in vitro* experiments with BMOs are more promising (118, 119).

Complex and hybrid *N*-glycans found in bovine mature milk and colostrum may also provide a source of prebiotic glycans that can be selectively utilized in a fashion similar to HMOs/BMOs (120). Further, the conjugation of these *N*-glycans to milk proteins enable different strategies for their recovery. Protein separation from lactose and subsequent treatment to separate *N*-glycans from their protein conjugates may offer a potentially attractive avenue to purification of these glycans (121). Thus, *N*-glycans derived from bovine colostrum, which is exceptionally rich in *N*-glycosylated proteins (122), may be a potent source of bioactive glycans to serve as prebiotic substrates. Extensive characterization of complex *N*-glycans derived from bovine milk proteins abundant in colostrum now shows that these *N*-glycans are highly selective for certain bacteria in the adult GI microbiome. The bacteria able to access these glycans are further restricted, relative to larger repeated polymers of less complex oligosaccharides which are limited to select *Bifidobacterium* species (e.g., *Bifidobacterium longum* subsp. *infantis*). Some strains of these species have been associated with diminished enteric inflammation and improved GI barrier function in humans (123, 124). Bovine colostrum is also a potential source of anti-infective glycans and recent work provides evidence for the anti-infective activity of oligosaccharides sourced from bovine colostrum against a highly invasive strain of *C. jejuni* (125).

## Clinical Applications of Bovine Colostrum

### Body Composition and Exercise Performance

The first study investigating the role of colostrum supplementation in exercise performance was completed in 1997 and showed marked improvements on explosive muscle power and increased concentration of immunoglobulins in serum (35). This finding is relevant given intense physical activity can suppress immunity several hours after training (126). Subsequent, well-controlled studies in comparison to whey protein concentrate have demonstrated significant improvements in lean body mass and weightlifting performance (127), in athletic performance among male and female athletes (128), speed in elite cyclists with dose-dependent effects (129), and in runners for recovery (130). Duff et al. (131) indicated that bovine colostrum supplementation (60 g/d of colostrum) on male and female older adults during resistance training is beneficial for increasing leg press strength and reducing bone resorption in comparison to whey protein complex supplementation. Improvement in the upper body strength, muscle thickness, lean tissue mass, and cognitive function were noted for colostrum supplemented group as well as whey protein treated group (131).

Despite this progress, the exact mechanism behind these marked improvements is not fully elucidated. As human studies typically use whey protein with similar protein content, observed differences are unlikely to simply be a response to protein digestibility or amino acid supplementation. Given that bovine colostrum immune components are likely not providing passive immunity to the human, it is possible that bioactive compounds and/or their metabolites have a direct effect on the immune system (35, 126, 132–135). There is currently weak support for the potential for bovine colostrum supplementation to improve leukocyte function relating to adaptive immunity (126). While a 33% increase in saliva IgA was noted after supplementation of colostrum at 20 g/day for 2 weeks (134) and a 79% increase in IgA in runners fed 12 g/day for 12 weeks was reported (132), these results were not repeated in other studies (35, 135–140). Further, colostrum supplementation diminished exercise-induced intestinal permeability which was replicated in *in vitro* culture models of intestinal epithelial cells (141). Considering the safety profile and generally positive past research from well-controlled studies, further research is warranted to understand the underlying mechanism and explain inter-individual variations and unexplored discrepancies between the growing number of studies on colostrum supplementation in regards to body composition and athletic performance.

### NSAIDs Induced GI Inflammation and Permeability

Non-steroidal anti-inflammatory drugs (NSAIDs) are the most common prescribed medicine and used for the symptomatic treatment of acute pain, chronic inflammatory, and degenerative joint diseases (142, 143). NSAIDs can cause gastric and intestinal damage such as peptic ulceration and injury to both the small and large intestine. Complications from NSAIDs use include increased intestinal permeability with protein and blood loss, and also stricture formation (142, 144). Approximately 2% of subjects taking NSAIDs experience adverse effects on the GI tract including bleeding, perforation, and inflammation. Acid suppressants and prostaglandin analogs are used to reduce gastric injury induced by NSAIDs, but these are not adequately effective in preventing small intestine injury. Hence, additional avenues for the mitigation of these negative side effects are needed. Some research suggests that colostrum may be an alternative, owing to the composition of growth factors like α-IGF-1, β-IGF-1, transforming growth factor (TGF), and epidermal growth factors (EGF). These growth factors are capable of stimulating the repair process of the GI tract (145) and are complementary to evidence supporting diminished GI permeability associated with exercise (141).

In a clinical examination of bovine colostrum for protection against NSAID-induced enteropathy, seven male volunteers (26–38 years old) who were taking NSAIDs or suffering from conditions likely to affect intestinal permeability (e.g., coeliac disease or previous intestinal surgery), were evaluated for the potential of bovine colostrum to alter intestinal permeability with concomitant indomethacin supplementation. In this crossover study, following an initial baseline permeability assessment, these volunteers were supplemented orally with 125 mL of bovine colostrum or a whey placebo three times daily for seven days. At the end of the trial period, intestinal permeability was reassessed and a 2-week “washout” period was performed between the crossover. Approximately a 3-fold increase in permeability was observed in the participants taking the whey placebo with indomethacin, while no significant increase in permeability was seen in the participants taking co-administration of bovine colostrum with indomethacin (146). In support of these findings, molecular characterization of the mechanism underlying these effects have been documented. Mir et al., (147) demonstrated that bovine lactoferrin can act as a carrier for NSAIDs by binding to these molecules, but with far lower affinity than the protein targets for NSAIDs which suggests that the efficacy of NSAIDs may not be affected by co-administration of a lactoferrin-containing protein source, like bovine colostrum (147). While further studies will be required to demonstrate that these compounds, when co-administered with bovine colostrum, maintain their desired efficacy, there is growing and consistent evidence supporting the potential for the use of bovine colostrum to manage the potential side effects of NSAIDs.

### Uses of Bovine Colostrum in Specific Clinical Populations

Bovine colostrum has led to human supplementation trials due to potential for improvement of GI health and integrity. Several conditions related to GI conditions associated with chronic or acute infections have been investigated for the potential of bovine colostrum to ameliorate symptoms associated with these conditions or infections. While the mechanisms behind these findings are difficult to disentangle given the disparate populations and disease etiologies, there are consistent themes related to the improvement of GI symptoms and reduced inflammation associated with each, though not all conditions demonstrate promising avenues for therapy.

#### Ulcerative Colitis

Ulcerative colitis, an inflammatory bowel disease associated with durable inflammation and ulcers in the colon (148), was investigated as a potential target for a bovine colostrum enema treatment in a small proof-of-concept trial. The authors rationalized this approach given the high concentrations of antimicrobial peptides, immunoglobulins and growth factors found in colostrum (149). In this pilot study, fourteen patients with active mild to moderate colitis were compared. Eight patients received 100 mL (10% solution) of bovine colostrum and six patients received an albumin placebo twice per day for 4 weeks. Improvement of the symptom score including patient well-being, abdominal pain, rectal bleeding, temperature, anorexia/nausea, bowel frequency, stool consistency, abdominal tenderness and the presence of extra-intestinal manifestations was reported in seven of the eight patients in the bovine colostrum treated groups (149). While this study is small, the findings show a significant reduction in symptom scores and follow up studies in a similar population with a larger sample size may be warranted.

#### Necrotizing Enterocolitis

Necrotizing enterocolisis (NEC) is one of the most common morbidities associated with preterm birth, and among the chief causes of mortality among infants born preterm (150). Several studies have examined the impact of either human or bovine-derived colostrum on NEC outcomes and development of preterm infants. In one clinical trial of 86 low birthweight infants supplemented with bovine colostrum in a dose of 2 g, four times per day for infants between 1,000 and 1,500 g and 1.2 g, four times per day for those under 1,000 g at birth. No significant differences were observed in the occurrence of NEC, sepsis, or mortality after the administration of bovine colostrum as compared with placebo (151). In a meta-analysis examining the use of bovine and human colostrum among preterm infants, Sadeghirad et al. concluded that the cumulative findings in the literature suggest that neither human nor bovine colostrum had an effect on the incidence of severe NEC, mortality, culture-proven sepsis, feed intolerance, or length (152). The lack of effects is observed on NEC patients due to some limitations such as using commercial bovine colostrum supplement and number of patients is modest (146, 147). Given these findings, it will be difficult to rationalize continued use of bovine or human colostrum with preterm infants for improvements in these outcomes. However, the use of human colostrum in preterm infants should not be curtailed based on these outcomes as other benefits have been demonstrated for preterm infants (153).

#### Traveler's Diarrhea

Acute infection with enterotoxigenic *Escherichia coli* (ETEC) represents the most common causes of so-called Traveler's Diarrhea, associated with travel to tropic and semitropical regions throughout the world (154). As bovine colostrum plays a key role in protecting the neonatal calf from environmental pathogens via passive immunity and ETEC represents one of the primary causative agents of neonatal calf diarrhea (155), researchers have been interested in determining whether the same effects can be demonstrated in humans at risk of Traveler's Diarrhea.

Using hyperimmune bovine colostrum which is rich in immunoglobulins targeting 14 strains of ETEC, the efficacy and dose response of consuming bovine colostrum in a tablet form (400 mg of bovine colostrum protein) demonstrated a dose-dependent and significant improvement in protecting against the development of diarrhea among volunteers in a double blinded, placebo-controlled ETEC challenge study. A 400 mg serving of hyperimmune bovine colostrum protein administered with a bicarbonate buffer three times daily conferred 90.9% protection when compared to the placebo (156). Bicarbonate buffer contributes to the enhancement the protective effects of hyperimmune colostrum protein in the ETEC challenge experiments, but the difference was not statistically significant. As little as 200 mg consumed three times per day without buffer gave an estimated 58.3% protection from diarrhea symptoms, compared to the placebo group (156).

In addition to ETEC, viruses contribute to a significant proportion of both neonatal calf diarrhea and Traveler's Diarrhea (154, 155). In a double blinded, placebo-controlled study, Mitra et al., (157) reported that consuming three daily servings of 100 mL of hyperimmune bovine colostrum targeting human rotavirus for 3 days conferred a modest but significant reduction in both the duration of diarrhea and the total stool output among male infants 6–24 months of age (157). Similarly, another study reported that purified immunoglobulins from hyperimmune bovine colostrum conferred a similar effect in acute rotavirus infection, supporting these findings (158).

While neither study examined the impact of colostrum from cows which had not been immunized against the target pathogen, a clinical trial examining the differences between hyperimmune bovine colostrum and bovine colostrum among children infected with shigellosis caused by *Shigella dysenteriae* (*S. dysenteriae*) failed to find any improvements among patients relative to the concurrent antibiotic therapy. However, preclinical studies in other biomedical models (e.g., gnotobiotic pigs) have shown promise for hyperimmune bovine colostrum in preventing diarrhea caused by *Clostridioides difficile* (*C. difficile*). Together, these findings may suggest that infectious mechanisms of pathogenesis shape the ability of hyperimmune bovine colostrum to influence disease progression as *S. dysenteriae* invades epithelial cells (159), potentially evading hyperimmune bovine colostrum immunoglobulins, while ETEC and *C. difficile* utilize secreted toxins to induce epithelial damage (160, 161).

## Future Directions

Given the biological role of colostrum for neonates (8, 162), its documented bioactive components as outlined here, and the potential for development as a functional food or food ingredient. There is a significant interest in developing colostrum as an ingredient to improve the bioactivity of foods and/or their potential health benefits. With a higher protein content and lower lactose concentration, this favorable protein/carbohydrate ratio is also nutritionally attractive and the potential for the future development of the ingredients and constituent fractions of colostrum is promising. However, overcoming processing challenges to separate bioactive fractions from colostrum remains a challenge to both study the mechanisms by which this fluid can act on humans and for practical product development. Future clinical trials should address current gaps in understanding which populations, such as those with GI disorders, may benefit most from colostrum consumption and whether whole or fractionated colostrum offers the most attractive balance of nutrition and bioactive properties.

## Author Contributions

SK organized the general content of the paper. AA was responsible for general editing and organizing the authors and also responsible for the two sections of the paper. MK contributed one section of the paper. HD was responsible for writing one section of the paper. AB was responsible for the one section of the paper. ME contributed to a section of the paper. BMH was responsible for the organizing a section. SAF contributed editing and organizing the paper. All authors contributed to the article and approved the submitted version.

## Conflict of Interest

BMH is an employee of Evolve Biosystems, Inc. SK has received funding from Uluova Süt Ticaret A.Ş (Uluova Milk Trading Co.), a company focused on the production of colostrum and lactoferrin. ME and AB are employees of Uluova Dairy. The remaining authors declare that the research was conducted in the absence of any commercial or financial relationships that could be construed as a potential conflict of interest.
